# Multicomponent Exercise Improves Physical Functioning but Not Cognition and Hemodynamic Parameters in Elderly Osteoarthritis Patients Regardless of Hypertension

**DOI:** 10.1155/2018/3714739

**Published:** 2018-03-12

**Authors:** Hélio José Coelho-Júnior, Ivan de Oliveira Gonçalvez, Iris Callado Sanches, Leandro Gonçalves, Erico Chagas Caperuto, Marco Carlos Uchida, Bruno Rodrigues

**Affiliations:** ^1^Applied Kinesiology Laboratory, School of Physical Education, Universidade de Campinas (UNICAMP), Campinas, SP, Brazil; ^2^Center of Health Sciences, Universidade de Mogi das Cruzes (UMC), Mogi das Cruzes, SP, Brazil; ^3^Human Movement Laboratory, São Judas Tadeu University (USJT), São Paulo, SP, Brazil

## Abstract

The present study aimed to investigate the impact of a 6-month multicomponent exercise program (MCEP) on physical function, cognition, and hemodynamic parameters of elderly normotensive (NTS) and hypertensive (HTS) osteoarthritis patients. A total of 99 elderly osteoarthritis patients (44 NTS and 55 HTS) were recruited and submitted to functional, cognitive, and hemodynamic evaluations before and after six months of a MCEP. The program of exercise was performed twice a week at moderate intensity. The physical exercises aggregated functional and walking exercises. Results indicate that 6 months of MCEP were able to improve one-leg stand and mobility (walking speeds) of osteoarthritis patients regardless of hypertension. On the other hand, cognitive and hemodynamic parameters were not altered after the MCEP. The findings of the present study demonstrate that 6 months of MCEP were able to improve the physical functioning (i.e., usual and maximal walking speed and balance) of osteoarthritis patients regardless of hypertensive condition.

## 1. Introduction

Osteoarthritis (OA) is the most common joint disorder in the world [1]. The incidence of OA increases according to aging, affecting more than 35% of the older adult population [1, 2]. The great concern regarding OA is its poor prognosis, since the progression of this disease collaborates with a severe impairment in physical functionality, reducing the capacity to perform the activities of daily living (ADL) and, consequently, the quality of life of elderly people [3, 4].

There is a growing body of evidence indicating that OA is associated not only with an impaired physical functioning but also with cardiovascular risk factors, such as hypertension [5–9]. In fact, data have demonstrated that hypertension and arterial stiffness, which have a key role in the genesis and progression of hypertension, stand out among the myriad of cardiovascular risk factors associated with OA (e.g., hyperinsulinemia, hyperglycemia, and low-grade inflammation) [6–9]. Taken together, these evidences may indicate a worse prognosis to hypertensive osteoarthritis (HTS-OA) patients due to the close relationship of OA and hypertension with numerous adverse outcomes.

It is worth mentioning that several findings have proposed that HTS patients can present impaired functional capacity, cognition, and adaptability in response to physical exercise when compared to normotensive (NTS) patients [5, 10–12]. On the other hand, HTS patients present larger reductions in blood pressure values after physical exercise programs compared to NTS patients [13, 14]. Therefore, it is possible to infer that HTS-OA patients can present an impaired adaptability in the physical and cognitive domains, while a large reduction in blood pressure is expected after physical exercise programs when these patients are compared to NTS-OA. However, this hypothesis has never been tested.

Physical exercise has been mentioned as a profitable nonpharmacological tool able to counteract the deleterious effects of OA and hypertension [13–15]. Recently, the American College of Sports and Medicine (ACSM) advises that physical exercise programs for health promotion should include different exercise regimes (e.g., aerobic, resistance, balance, and flexibility) in an attempt to offer a large number of stimuli, probably causing superior beneficial effects [16, 17]. Multicomponent exercise program (MCEP) emerges as a kind of exercise able to contemplate ACSM recommendations because its design allows the performance of different modalities of exercise (e.g., aerobic, resistance, stretching, and balance) mixed in the same exercise session or routine [18].

Evidence has demonstrated beneficial effects of MCEP on the physical functioning and cognitive parameters of OA patients [19–21], and similar findings have been observed in HTS patients [20]. However, the experiments were designed based on low sample sizes, short periods of intervention (i.e., 3 months), and subjective methods for physical evaluation (e.g., questionnaires), limiting better inferences. Furthermore, to the best of our knowledge, the effects of a MCEP on physical functioning, cognition, and hemodynamic parameters of HTS-OA patients have not been elucidated. Lastly, there is no evidence exploring if hypertension may impair the adaptability of OA patients in response to physical exercise.

Therefore, the present study aimed to investigate the impact of a 6-month MCEP on physical functioning, cognition, and hemodynamic parameters of OA patients. Moreover, we investigated if hypertension could impair the adaptability of OA patients in response to physical exercise.

## 2. Methods

### 2.1. Design

**Figure 1:**
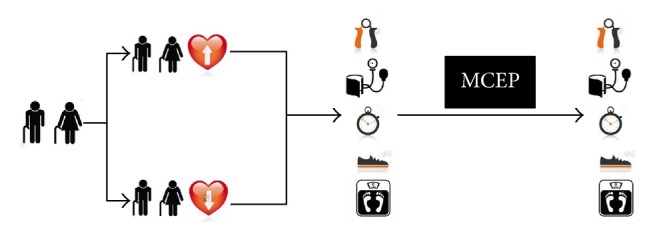
The experimental design used in the present study.

The present investigation has a quasi-experimental design, which aimed to determine the effects of a 6-month MCEP on functional, cognitive, and hemodynamic parameters of normotensive (NTS) and hypertensive (HTS) elderly patients with lower limb osteoarthritis (OA) (Figure 1). Therefore, patients were submitted to functional, cognitive, and hemodynamic evaluations before and after 6 months of a MCEP. Experiments were developed in the city of Poá, state of São Paulo, Brazil, in 2016.

### 2.2. Subjects

Initially, 99 elderly volunteers, clinically diagnosed with lower limb OA, were recruited by convenience from two specialized healthcare centers for older adults in a town located in the metropolitan area of São Paulo, Brazil. Subsequently, two groups (NTS-OA [*n* = 44] and HTS-OA [*n* = 55]) were divided from the initial sample based on the diagnosis of hypertension.

Eligibility criteria for this study were based on the presence of a clinical diagnosis of lower limb OA, hypertension (to HTS-OA group), and age ≥60 years. Patients of both sexes were accepted in the study. Patients who presented changes of antihypertensive medication during the study, missing values, physical (e.g., angina) and/or psychological (e.g., fear) discomfort during exercise sessions, cerebrovascular disease (e.g., stroke), pulmonary disease, neurological or psychiatric disease (e.g., Parkinson's or Alzheimer's disease), musculoskeletal disorders, chronic rheumatic condition other than OA, allocation for arthroplasty (i.e., end-stage hip and knee OA), comorbidities associated with greater risk of falls and any kind of dizziness, and blurred vision or lightheadedness when rising or remaining standing for long, which could indicate orthostatic hypotension and/or labyrinthitis, were absent from more than three sessions of physical exercise, and did not complete the entire battery of evaluations were excluded. We also excluded participants who were prescribed hormone replacement therapy and/or psychotropic drugs. It is worth mentioning that the volunteers were not under the use of medications to treat symptoms of OA, only hypertension (HTS-OA group). However, they reported making occasional (once every 15–30 days) use of analgesics, anti-inflammatories, and/or muscle relaxants. Therefore, we excluded volunteers that started a chronic pharmacological treatment for OA symptoms for the duration of the study. The use of medications and exclusion criteria data were collected from medical records (chart review) of each subject. In addition, since OA patients may present pain, muscle fatigue, or even low muscle strength during the performance of the tests, the time to perform the test was not an exclusion criterion, given that the volunteer could take as long as necessary to perform the test. Lastly, volunteers that scored 0 in the one-leg stand test were not excluded from the present study, if they performed the other tests. This occurred because a null result in this test indicates a low physical performance and not a missing value.

All volunteers signed the informed consent form and completed all measurements. This study was approved by the Research Ethics Committee of the University of Campinas (UNICAMP) under protocol number 835.733. This study was developed in accordance with the Declaration of Helsinki and according to Resolution 196/96 of the National Health Council.

Since both healthcare complexes serve a large number of patients and the medical team (i.e., nurse, physician, and physical educator) is of limited size, the pathological conditions were simply recorded by the head physician and head nurse of each center. A specialist (i.e., rheumatologist and cardiologist) who was not affiliated to and was outside the center then made the diagnosis of OA and/or hypertension, according to the specific guidelines of each disease (i.e., American College of Rheumatology [ACR] [22, 23] and Brazilian Society of Cardiology [BSC] [24], resp.).

### 2.3. Evaluations

All volunteers were instructed to refrain from any exhausting physical activity for a period of 96 h earlier and drinking alcoholic and caffeinated beverages 24 h before testing. Although alimentary ingestion was not controlled, subjects were instructed to maintain their food intake during the study period. Baseline evaluations (i.e., pre) were performed 5 days (i.e., 120 hours) before the beginning of the MCEP. Likewise, the final evaluations were performed on the fifth day after the last exercise session. The protocol used for morphological, functional, cognitive, and hemodynamic evaluations was mentioned by our group elsewhere [10, 11].

### 2.4. Morphological Measurements

A body weight scale with stadiometer Filizola® (Brazil) was used for weight (kg) and height (cm) measurements. An anthropometric tape (flexible and inextensible) Sanny® (Brazil) was used to measure waist (WC), hip (HC), and neck (NC) circumferences. For evaluations, subjects wore light clothing, in the standing position, with head held erect and eyes forward, with the arms relaxed at the side and feet in parallel (i.e., together). The WC was evaluated at the midpoint between the last floating rib and the highest point of the iliac crest. HC was evaluated at the highest point of the buttocks. NC was measured at the height of the gland cricoid cartilage prominence. All the subjects were evaluated twice, and the highest value was used for analysis.

### 2.5. Functional Evaluations

Two experienced researchers applied each test. While one was responsible for detailing the operational procedures, demonstrating the test before the evaluation, quantifying the evaluation time, and evaluating the motor gesture, the other ensured the safety of the participant. After the end of the explanation and before the start of the tests, volunteers performed a familiarization trial to ensure the understanding of the test. Then, the volunteers performed all tests twice, and the best result obtained in each test was used in the analysis. The tests were distributed in a room as stations and were performed in a circuited fashion one after the other. A one-minute interval between trials was provided. During all tests, verbal encouragement was provided to ensure that volunteers achieved the best possible performance without compromising safety. During TUG, walking speed test at maximal pace, and sit-to-stand tests, researchers provided stimulus such as* come on, faster!*;* a little more!*; and* let's go! *During OLS, verbal encouragement was provided to keep the participant focused on the test. Therefore, the volunteers were stimulated with the following sentences:* focus!*;* keep your posture!*; and* very good!* During handgrip test, the researchers repeatedly used the following sentences:* as much force as possible!; let's go!; *and* more strength! *For the countermovement jump test, verbal encouragement was only provided before the test, with the following sentence:* jump as high as you can using all your strength! *Regarding six-minute walk test, researchers told the volunteers that they were close to finalizing the test (i.e.,* come on!; force!; there is little left!*).

#### 2.5.1. Sit-to-Stand Test

Volunteers were requested to rise from a chair five times as quickly as possible with arms folded across the chest. The stopwatch was activated when the volunteer raised their buttocks off the chair and was stopped when the volunteer seated back at the end of the fifth stand.

#### 2.5.2. One-Leg Stand Test

The one-leg stand test was performed with the volunteers standing in a unipodal stance with the dominant lower limb, the contralateral knee remaining flexed at 90°, the arms folded across the chest, and the head straight. A stopwatch was activated when the volunteer raised their foot off the floor and was stopped when the foot touched the floor again. The maximum performance time was up to 30 seconds, considered the best test result.

#### 2.5.3. Walking Speed Test (Usual and Maximal)

Walking speed was measured over three meters. This distance was chosen due to space limitations. It is worth mentioning that a high concordance has been observed between the results recorded after 3-meter and 6-meter courses [25]. In the test, volunteers were required to walk five meters at their usual and fastest possible cadences (without running). Before the evaluation, both feet of each volunteer were to remain on the starting line. The measurement was started when a foot reached the one-meter line and was stopped when a foot reached the four-meter line. The one-meter intervals at the beginning and at the end of the course were used to avoid early acceleration and/or deceleration.

#### 2.5.4. Timed “Up and Go” (TUG) Test

The TUG test involves getting up from a chair (total height: 87 cm; seat height: 45 cm; width: 33 cm;), walking three meters around a marker placed on the floor, coming back to the same position, and sitting back on the chair. The subjects who started the test wore their regular footwear, with their back against the chair, arms resting on the chair's arms, and the feet in contact with the ground. A researcher instructed the volunteers, on the word “go,” to get up and walk as fast as possible without compromising safety in the demarcation of three meters on the ground, turn, return to the chair, and sit down again. Timing was started when the volunteer got up from the chair and was stopped when the participant's back touches the backrest of the chair. A stopwatch (1/100 second accuracy) was used for time evaluation, and a longer time taken to perform the test indicates a lower performance.

### 2.6. Cognitive Evaluation: Executive Function (EF)

#### 2.6.1. TUG Cognitive Test

TUG cognitive test was accomplished to evaluate EF. This test is performed as the conventional TUG; however, a cognitive task (verbal fluency, animal category) should be accomplished during the motor task. Therefore, after the signal of the evaluator, volunteer performed the route—stand up from the chair, walk three meters, turn around, walk three meters back, and sit down again—naming as many animals as they could remember. This task should be performed aloud, allowing the evaluators to confirm if the volunteers were, in fact, accomplishing the task. The time expanded to perform the task was recorded for evaluation [26].

### 2.7. Hemodynamic Measurements

The procedures for measurement of blood pressure were adapted from the VII Joint National Committee on Prevention, Detection, Evaluation, and Treatment of High Blood Pressure (JNC7) [27]. In summary, patients remained in a sitting position on a comfortable chair for 15 minutes in a quiet room. After this period, an appropriate cuff was placed at approximately the midpoint of the upper left arm (heart level). An automatic, noninvasive, and validated [28] arterial blood pressure monitor (Microlife-BP 3BT0A, Microlife, Widnau, Switzerland) was used to measure systolic blood pressure (SBP), diastolic blood pressure (DBP), and heart rate (HR). During blood pressure recording, volunteers remained relaxed in the sitting position, with parallel feet at one shoulder width, both forearm and hands on the table, supinated hands, backs against the chair, without move or talk. The volunteer did not have access to blood pressure values during measurement. The evaluation lasted approximately 80 seconds and was performed three times with one minute of rest among the measurements. The mean of measurements of each volunteer was used in the final analysis. Mean arterial pressure (MAP), double product (DP), and pulse pressure (PP) were evaluated according to the following equations: MAP = [SBP + (2*∗*DBP)]/3, DP = SBP *∗* HR, and PP = SBP − DBP. The size of the arm cuff was selected after measuring the arm circumference of each participant (Sanny, São Paulo, Brazil). All volunteers were evaluated within the first two months after the update of the medical records.

### 2.8. Multicomponent Exercise Program (MCEP)

The MCEP was performed twice a week, on nonconsecutive days, during 26 weeks at the fitness center of an institutional center for elderly care and living (Centro de Convivência do Idoso [CCI]), Poá, Brazil. The program was designed to offer exercises that would mimic activities of daily life (ADL) gestures, thereby inducing neuromuscular adaptations to keep the subjects capable of performing the ADL. Each exercise session was composed of 12 different exercise stations. Each exercise session structure was defined by the sequence of one functional exercise followed immediately by a brief walk. Exercise session was composed of approximately 12 minutes of functional exercises, 24 minutes of walk, and 12 minutes of rest. Each session of exercise was composed of approximately 50 patients. A professional physical trainer with long experience in exercise training with elderly people supervised all sessions. Volunteers were instructed to avoid the Valsalva maneuver during the performance of exercises.

The functional exercises were changed during the whole program. However, they always represented ADL with a high necessity of the activity of the lower limbs, for example, stand up and sit on a chair, pick up a weight off the floor and put it on top of a structure, and transfer a weight from one place to another. Balance and proprioception exercises also comprised functional exercises, as one-leg stand. At most, three balance and/or proprioception exercises were used in each session. To complete the list of physical exercises, upper limbs resistance exercises were added.

All functional exercises were performed for one minute. The brief walk was performed for two minutes. Thus, after the end of each functional exercise, volunteers should walk from one point to another (30 m), around a cone, come back to the initial line (30 m), and start the path again until completing the two minutes. A rest interval of 60 seconds was adopted between the stations [11].

### 2.9. Exercise Intensity Control

The control of exercise intensity was accomplished by the rating of perceived exertion (RPE) method using the adapted Borg scale (2001) (i.e., CR-10) [29], which was used to ensure that volunteers performed the exercises in the desired intensity. This scale is composed of eleven numbers (i.e., 0, 1, 2, 3, 4,…) and eight descriptors (i.e., rest; very, very easy; easy; moderate; somewhat hard; hard; very hard; and maximal), which represents the perception of effort of the subject in front of an exercise load. The higher the reported number, the greater the sensation of effort [29]. During the performance of functional—except for balance exercises—and resistance exercises, volunteers were instructed to maintain the physical activity intensity in 3–5, which represents moderate (i.e., 3), somewhat hard (i.e., 4), and hard (i.e., 5) descriptors. For that, a large picture of RPE scale (i.e., 4 meters high and 1.30 meters wide) was positioned on the wall in the gym's room. The increase in the exercise intensity was based on alterations in the cadence of the performance (i.e., faster) for functional exercises and walk. Moreover, for resistance exercises, volunteers could use elastic bands (EXTEX Sports, São Paulo, Brazil) and dumbbells to reach the intensity prescribed.

### 2.10. Statistical Analyses

Normality of data was tested using the D'Agostino-Pearson omnibus normality test. Student's* t*-test for independent samples and Mann–Whitney test were used for comparisons between the groups (unpaired) for parametric and nonparametric samples, respectively. Student's* t*-test for dependent samples and Wilcoxon's signed-rank test were used for intragroup comparisons (paired) for parametric and nonparametric samples, respectively. Cohen's effect size *d* was calculated to assess the magnitude of the results. The effect size was classified according to Rhea (2004) [30]. The level of significance was 5% (*P* < 0.05) and all procedures were performed using the Statistical Package for the Social Sciences software (New York, USA).

## 3. Results

No adverse events occurred during the sessions of exercise or during any of the evaluations. The subjects were not absent for more than three sessions of physical exercise. Adherence to the physical exercise program was 100% (0 dropouts). Volunteers did not report any changes in food intake and in the number/class of medications during the study.

Table 1 shows the main characteristics of NTS-OA and HTS-OA. It is possible to observe that NTS and HTS volunteers presented an overweight phenotype (BMI ≥ 25 kg/m^2^). The analysis of circumferences is in congruence with BMI results and indicates that the volunteers were at a moderate-to-high cardiovascular risk. In relation to sex, our sample presented high prevalence of older women when compared to older men. This pattern is in concordance with previous findings of global burden of diseases studies in which higher prevalence of lower-limb OA was observed in women than in men regardless of the site affected by the disease (i.e., knee or hip) [31, 32]. Although authors have not proposed the main mechanisms responsible for this phenomenon, the marked decrease in sex hormones observed during menopause, primarily oestrogen, has been considered a possible factor to explain the higher predisposition presented by older women to OA when compared to older men [33, 34]. The mean of medications was 1.4 per volunteer, considering that 3.6% utilized ≥3 medications, 29.1% used 2 medications, and 67.3 used only one medication. Diuretic (69.1%) was the most prevalent class of antihypertensive medication, followed by beta-blocker (34.5%), angiotensin-II receptor antagonist (16.4%), angiotensin-converting enzyme inhibitor (9.1%), and calcium channel blockers (3.6%).

Hypothesis test showed that HTS-OA presented higher body mass, BMI, WC, HC, NC, SBP, and HR when compared to NTS-OA.

Table 2 shows the anthropometric parameters. MCEP was not effective to cause significant changes in anthropometric parameters of NTS-OA or HTS-OA. In addition, the magnitude of alterations (Δ [%]) was not different between the groups.

Cognitive and functional parameters are shown in Table 3. A significant increase in one-leg stand (NTS-OA = +101.9%; HTS-OA = +107.5%) and maximal walking speed (NTS-OA = +253.7%; HTS-OA = +270.0%) was observed in NTS-OA and HTS-OA patients. However, only HTS-OA presented a significant increase in usual walking speed performance (+18.8%). Exercise training did not cause significant improvements in sit-to-stand, TUG, or TUG exercise with a cognitive task. The magnitudes of changes (Δ [%]) in the functional and cognitive parameters were similar in the groups after the MCEP.

Table 4 shows the hemodynamic parameters. NTS-OA did not present significant alteration in any of the hemodynamic parameters. On the other hand, HTS-OA presented a significant increase in HR.

## 4. Discussion

The main findings of the current study indicate that a 6-month MCEP is able to improve physical functioning of OA patients. However, contrary to our hypothesis, the cognitive and hemodynamic parameters were not altered after the MCEP, thereby indicating that HTS condition did not impair the adaptability of OA patients to physical exercise.

Just a few studies have explored the effects of MCEPs on physical function of samples composed exclusively of elderly OA patients. In addition, most of these investigations have used subjective methods (i.e., self-rated questionnaires) to measure physical function, which may not reflect the real state of the evaluated parameter [15]. As for studies that directly assessed physical function, Levy et al. [20] observed an increased transfer capacity (i.e., 8-foot up and go) in older adults with OA after 3 months of MCEP. Similarly, middle-aged and older adults with OA from the study of Ağlamış et al. [19] presented an improved lower-body muscle strength (i.e., chair stand) in response to a 3-month MCEP.

These results differ from the present study, where transfer capacity—evaluated by TUG test—and lower-body muscle strength—evaluated by chair stand test—did not change after 6 months of MCEP. It is worth mentioning that MCEPs are characterized by a session of exercise that comprises a mix of different exercise regimes, allowing different designs of MCEP, which can explain most of the different results observed between the studies. In fact, it is possible to observe that Levy et al. [20] proposed a MCEP composed of aerobic, resistance, balance, core strength, calisthenics, flexibility, dance, and muscle power exercises.

Interestingly, the evaluation of transfer capacity is strongly associated with muscle power [35, 36], and improvements in this physical capacity are stimulus-dependent (i.e., higher velocity concentric muscle contractions) [37]. However, different from Levy et al. [20], the current MCEP was not composed of muscle power exercises, and this feature may be indicated as the main factor responsible for the different results observed between the trials. However, Levy et al. [20] offer a poor description about their MCEP, because the variables associated with exercise prescription (e.g., intensity, volume, density) were absent in the materials and methods section, limiting its external validity and better comparisons between the studies.

In relation to Ağlamış et al. [19], the authors proposed a MCEP composed of a progressive strength training component, characterized by lower-limb and respiratory exercises performed in a circuit fashion, where each exercise was repeated 3 times (i.e., 3 sets). In the current study, each functional and resistance exercise was performed just once, and findings from original studies and meta-analytic regression have demonstrated that multiple-set resistance training programs are superior to single-set resistance training programs to elicit increase in muscle strength [38, 39]. Therefore, it is possible to infer that the differences between the studies regarding the improvements in lower-limb muscle strength are the product of an insufficient resistance training program offered by us in our MCEP.

However, it should be stressed that our MCEP was designed to be offered in public health programs that have to deal with a large number of patients, making it difficult to prescribe such exercise. Therefore, although the performance of a progressive resistance training with optimal volume (~3 sets per exercise) seems to be the ideal approach to improve the neuromuscular function of older adults [16, 40], it does not fit into MCEP targeting public health programs.

In addition to the aforementioned findings, we observed significant increases in balance (i.e., one-leg stand test) and mobility (i.e., usual and maximal walking speed tests) after the MCEP. These results have large external validity because these physical capabilities are predictors of adverse health-related events in elderly adults. Indeed, balance is a well-known predictor of falls, since balance disorders account for 17% of the causes of fall in elderly adults [41]. In relation to mobility, walking speed reflects the functioning of several body systems, including but not limited to the cardiovascular, musculoskeletal, respiratory, and neural systems [42]. In addition, several observational studies have demonstrated that a limited walking speed is associated with adverse outcomes, such as lower extremity limitations, difficulty or inability to perform the ADL, cognitive impairment, falls, and mortality [42–44]. Lastly, walking speed test is a diagnostic tool in the context of sarcopenia [45].

Regarding the cognitive parameters, EF was selected as our cognitive assessment, since it aggregates several cognitive abilities, such as planning, problem-solving, identification, observation, conclusions about the outcomes, and inhibition of influencing factors [46].

Although in the literature there is a lack of specific studies investigating the behavior of EF in elderly OA patients, several findings describe that the aging process is strongly associated with executive dysfunction [26]. Furthermore, just a few investigations have evaluated the effect of MCEPs on cognitive domains of older adults [47, 48] and, to the best of our knowledge, just one study investigated OA patients [19].

In this study, Ağlamış et al. [19] observed a significant increase in the mental health of OA patients after the MCEP. However, the cognitive evaluation was based on the SF-36 health survey questionnaire, limiting the discussion. Nevertheless, it is important to mention that the effects of exercise training on the cognitive domains are still unclear, making inferences impossible. Therefore, more investigations about the effects of different MCEP on cognitive domains of OA are still necessary.

As aforementioned, there is a growing number of evidences indicating that OA patients present high prevalence of cardiovascular risk factors, including high blood pressure [5–8]. Nevertheless, for the first time in literature, we tested the hypothesis* (H1)* that multicomponent exercise could elicit a significant decrease in blood pressure values of OA patients. However, confirming the null hypothesis* (HO)*, the hemodynamic parameters of OA patients were not different in the periods before and after exercise regardless of hypertension.

Although the molecular pathways responsible for this link are mostly unknown, several mechanisms have been proposed to be responsible for this phenomenon, such as chronic low-grade inflammation, aging, obesity, and medications [7]. Regarding low-grade inflammation, authors have described that this state occurs in response to repeated knee trauma and biomechanical overloading in OA patients [7]. This seems to be plausible because OA grade is associated with arterial stiffness and matrix metalloproteinase-3 (MMP3) [8], which are activated by an inflammatory state [7].

Furthermore, the proinflammatory cytokines elicit the production of reactive oxygen species (ROS) that act in the development of endothelial dysfunction, decreasing the bioavailability of vasodilatory substances (e.g., nitric oxide [NO]) [49].

It is noteworthy that investigations [50, 51] that approached a resistance component performed at higher intensities than was used in the current study observed a significant decrease in blood pressure values, which was associated with significant alterations in inflammatory markers (i.e., C-reactive protein and TNF-*α*), NO bioavailability, and fat mass. Regarding the aerobic component, studies using a larger frequency, volume, and intensity when compared to our MCEP have demonstrated regulation of ROS production and an increase in NO levels [52, 53]. Therefore, it is possible that the exercise regimes (e.g., resistance and aerobic) mixed in our MCEP were not able to reverse the aforementioned environment probably present in HTS-OA patients (e.g., endothelial dysfunction and decreased NO bioavailability), consequently failing to activate the physiological mechanisms necessary to cause reductions in blood pressure.

In addition, findings have been discussing that HTS patients may present an impaired adaptability to physical stimulus when compared to NTS patients. In fact, evidences have discussed a possible association between hypertension and low physical capacity and cognition [5, 10–12]. However, the findings have demonstrated conflicting results. The current study adds evidence to previous studies [5, 10–12] and indicates that hypertension did not impair the adaptability to physical exercise.

Furthermore, the magnitude of changes in blood pressure values after aerobic and/or resistance exercise seems to be dependent on preexercise values, since HTS patients show a larger decrease in blood pressure after exercise when compared to NTS patients [13, 14]. However, in the present study, data did not confirm previous investigations and indicate that hypertension did not alter the magnitude of changes in blood pressure values of OA patients after MCEP.

Some limitations of the present study should be mentioned and addressed in future studies for a better understanding about the effects of MCEP in OA patients, such as the lack of information regarding the educational level of the volunteers, blood analyses of the participants, evaluation of other cognitive domains, other designs of multicomponent exercise, a sedentary CG, and information regarding OA severity. Regarding the latter, it should be stressed that our volunteers were only clinically classified, and the severity of OA was not considered in the analysis. Therefore, future studies should investigate the relation of the results of the present study to the extent of OA. In addition, it is possible to observe high prevalence of elderly women in our sample. In an attempt to observe the effects of sex bias on the present study, data were reanalyzed based only on female volunteers (Supplementary Material [SM]; Figures SM1 and SM2). It is worth mentioning that similar results were observed in elderly women when compared to the total sample. Therefore, future studies should verify if these data are replicated in samples composed only of men.

## 5. Conclusion

In conclusion, data from the present study demonstrate that 6 months of MCEP were able to improve physical functioning (i.e., balance and mobility) of OA patients regardless of hypertensive condition.

## Figures and Tables

**Table 1:** Comparison between the groups regarding the morphological and hemodynamic parameters.

Variables	NTS-OA (*n* = 44)	HTS-OA (*n* = 55)
*Subjects' characteristics*		
Age (years)	63.9 ± 3.7 (60–71)	68.4 ± 6.4 (60–85)
Weight (kg)	66.6 ± 11.8 (48.1–89.8)	73.7 ± 14.1 (44–109)^*∗*^
Height (cm)	154.5 ± 2.5 (1.5–1.9)	160.1 ± 6.0 (1.4–1.7)
BMI (kg/m^2^)	26.6 ± 4.3 (16.6–39.7)	28.8 ± 5.7 (20.3–43.4)^*∗*^
WC (cm)	92.5 ± 12.4 (63–124)	100.5 ± 13.3 (72–133)^*∗*^
HC (cm)	102.5 ± 8.1 (88–120)	107.6 ± 12.1 (89–141)^*∗*^
NC (cm)	35.4 ± 2.9 (30–45)	36.8 ± 3.2 (30–44)^*∗*^
Knee OA (%)	5.3	18.2
Hip OA (%)	68.2	69.1
Knee and hip OA (%)	40.0	20.0
Female (%)	92.3	90.9
Mean of medication	—	1.4 ± 1.4
*Drug class (%)*		
Diuretic	—	69.1
Beta-blocker	—	34.5
ANG-II receptor antagonist	—	16.4
ACE inhibitor	—	9.1
Calcium channel blockers	—	3.6
*Hemodynamic parameters*		
SBP (mmHg)	127.6 ± 14.6 (96–162)	135.6 ± 18.2 (99–181)^*∗*^
DBP (mmHg)	76.3 ± 9.7 (58–97)	76.8 ± 11.9 (58–109)
MAP (mmHg)	89.2 ± 21.9 (73–114)	94.7 ± 17.7 (78–123)
HR (bpm)	79.3 ± 10.0 (53–108)	74.2 ± 11.3 (35–113)^*∗*^
DP (mmHg·bpm)	9693 ± 2906 (6625–17496)	9930 ± 2587 (4270–14238)
PP (mmHg)	48.9 ± 16.7 (18–79)	57.6 ± 17.8 (30–97)

Data are presented as mean ± SD (min–max). NTS: normotensive; HTS: hypertensive; SBP: systolic blood pressure; DBP: diastolic blood pressure; MAP: mean arterial pressure; HR: heart rate; DP: double product; PP: pulse pressure; ^*∗*^*P* < 0.05 versus NG; ANG: angiotensin; ACE: angiotensin-converting enzyme.

**Table 2:** Effect of MEP on anthropometric parameters.

Variable	NTS-OA	HTS-OA
Body mass (kg)		
Pre	66.6 ± 11.8 (48.1–89.8)	73.7 ± 14.1 (44–109)
Post	65.3 ± 11.9 (49–106.6)	74.9 ± 13.9 (44–105)
Δ (%)	−0.79	6.51
ES	0.10 (trivial)	−0.08 (trivial)
Height (cm)		
Pre	154.5 ± 2.5 (1.5–1.9)	160.1 ± 6.0 (1.4–1.7)
Post	153.5 ± 5.8 (1.4–1.9)	157.0 ± 6.8 (1.4–1.8)
Δ (%)	−2.6	−1.7
ES	0.22 (trivial)	0.48 (trivial)
BMI (kg/m^2^)		
Pre	26.6 ± 4.3 (16.6–39.7)	28.8 ± 5.7 (20.3–43.4)
Post	27.8 ± 4.3 (20.5–42.7)	30.4 ± 5.8 (19–44.5)
Δ (%)	7.0	10.4
ES	−0.27 (trivial)	−0.27 (trivial)
Waist circumference (cm)		
Pre	92.5 ± 12.4 (63–124)	100.5 ± 13.3 (72–133)
Post	94.2 ± 10.7 (74–114)	100.3 ± 19.4 (71–144)
Δ (%)	3.6	1.6
ES	−0.14 (trivial)	0.01 (trivial)
Hip circumference (cm)		
Pre	102.5 ± 8.1 (88–120)	107.6 ± 12.1 (89–141)
Post	101.6 ± 10.3 (64–129)	104.1 ± 18.1 (87–143)
Δ (%)	−0.1	−1.8
ES	0.09 (trivial)	0.22 (trivial)
Neck circumference (cm)		
Pre	35.4 ± 2.9 (30–45)	36.8 ± 3.2 (30–44)
Post	35.2 ± 5.5 (30–41)	37.7 ± 8.4 (32–43)
Δ (%)	−0.2	3.2
ES	0.04 (trivial)	−0.14 (trivial)

Data are presented as mean ± SD (min–max); NTS: normotensive; HTS: hypertensive; BMI: body mass index; ES: effect size (min–max).

**Table 3:** Effect of MEP on functional parameters.

Variable	NTS-OA	HTS-OA
Sit-to-stand (s)		
Pre	11.1 ± 3.4 (8–20.7)	10.6 ± 2.6 (5–16.7)
Post	10.8 ± 3.1 (6.3–20.5)	10.4 ± 2.6 (5–16.9)
Δ (%)	−1.1	6.1
ES	0.09 (trivial)	0.07 (trivial)
One-leg stand (s)		
Pre	18.3 ± 13.4 (0–30)	15.6 ± 11.4 (0–30)
Post	24.0 ± 8.3 (0–30)^*α*^	21.8 ± 9.1 (0–30)^*α*^
Δ (%)	101.9	107.5
ES	−0.51 (small)	−0.60 (small)
Usual walking speed (m/s)		
Pre	0.82 ± 0.20 (0.5–1.7)	0.90 ± 0.22 (0.5–1.7)
Post	2.19 ± 0.50 (1.3–3.6)	2.29 ± 0.60 (0.4–1.3)^*α*^
Δ (%)	−7.8	−10.6
ES	0.34 (trivial)	0.69 (small)
Maximal walking speed (m/s)		
Pre	0.91 ± 0.30 (0.5–2)	0.94 ± 0.30 (0.6–1.8)
Post	1.73 ± 0.30 (1.2–2.5)^*α*^	1.74 ± 0.40 (1.2–1.8)^*α*^
Δ (%)	−70.7	71.4
ES	2.75 (large)	3.81 (large)
TUG (s)		
Pre	6.53 ± 1.52 (5.1–10.9)	7.34 ± 1.26 (5.4–10.3)
Post	6.86 ± 1.23 (5–10.9)	7.05 ± 2.04 (5–13.6)
Δ (%)	4.3	−2.2
ES	−0.23 (trivial)	0.17 (trivial)
TUG with a cognitive task (s)		
Pre	7.41 ± 1.50 (4.7–12.6)	8.30 ± 1.96 (5.7–16.9)
Post	7.91 ± 1.69 (5–11.9)	8.43 ± 2.57 (5–17.9)
Δ (%)	11.0	4.8
ES	−0.31 (trivial)	−0.05 (trivial)

Data are presented as mean ± SD (min–max); NTS: normotensive; HTS: hypertensive; TUG: timed up and go; ES: effect size; ^*α*^*P* < 0.05 versus pre.

**Table 4:** Effect of MEP on hemodynamic parameters.

Variable	NTS-OA	HTS-OA
SBP (mmHg)		
Pre	127.6 ± 14.6 (96–162)	135.6 ± 18.2 (99–181)
Post	128.3 ± 17.8 (94–178)	138.9 ± 20.9 (87–196)
Δ (%)	2.2	2.0
ES	−0.04 (trivial)	−0.16 (trivial)
DBP (mmHg)		
Pre	76.3 ± 9.7 (58–97)	76.8 ± 11.9 (58–109)
Post	73.8 ± 11.4 (49–113)	78.0 ± 15.0 (59–149)
Δ (%)	−1.9	1.6
ES	0.23 (trivial)	−0.08 (trivial)
MAP (mmHg)		
Pre	89.2 ± 21.9 (73–114)	94.7 ± 17.7 (78–123)
Post	91.9 ± 12.5 (65–125)	96.2 ± 20.4 (69–196)
Δ (%)	−0.3	1.2
ES	−0.15 (trivial)	−0.07 (trivial)
HR (bpm)		
Pre	79.3 ± 10.0 (53–108)	74.2 ± 11.3 (35–113)
Post	79.6 ± 10.2 (51–102)	78.6 ± 11.3 (58–130)^*α*^
Δ (%)	2.2	2.3
ES	−0.02 (trivial)	−0.38 (trivial)
DP (mmHg·bpm)		
Pre	9693 ± 2906 (6625–17496)	9930 ± 2587 (4270–14238)
Post	10206 ± 1869 (4794–14596)	10530 ± 3194 (6438–17640)
Δ (%)	4.5	6.6
ES	−0.20 (trivial)	−0.20 (trivial)
PP		
Pre	48.9 ± 16.7 (18–79)	57.6 ± 17.8 (30–97)
Post	54.3 ± 13.5 (18–81)	59.6 ± 16.8 (27–99)
Δ (%)	16.8	10.5
ES	−0.35 (trivial)	−0.11 (trivial)

Data are presented as mean ± SD (min–max); NTS: normotensive; SBP: systolic blood pressure; DBP: diastolic blood pressure; MAP: mean arterial pressure; HR: heart rate; DP: double product; PP: pulse pressure; ^*α*^*P* < 0.05 versus pre.

## Supplementary Materials

Supplementary Materials Supplementary Materials may be observed in Figures SM1 and SM2. Figure SM1: functional and cognitive parameters. Data are presented as mean ± SD; NTS: normotensive; HTS: hypertensive; TUG: timed up and go; *αP* < 0.05 versus pre. Figure SM2: hemodynamic parameters. Data are presented as mean ± SD; NTS: normotensive; HTS: hypertensive; SBP: systolic blood pressure; DBP: diastolic blood pressure; MAP: mean arterial pressure; HR: heart rate; DP: double product; PP: pulse pressure; *αP* < 0.05 versus pre.
