# Supplementary figures and images for: Multicomponent Exercise Improves Physical Functioning but Not Cognition and Hemodynamic Parameters in Elderly Osteoarthritis Patients Regardless of Hypertension
Source: Biomed Res Int. 2018 Mar 12;2018:3714739. doi: 10.1155/2018/3714739 (PMC5867618; doi:10.1155/2018/3714739)

## Slide 1
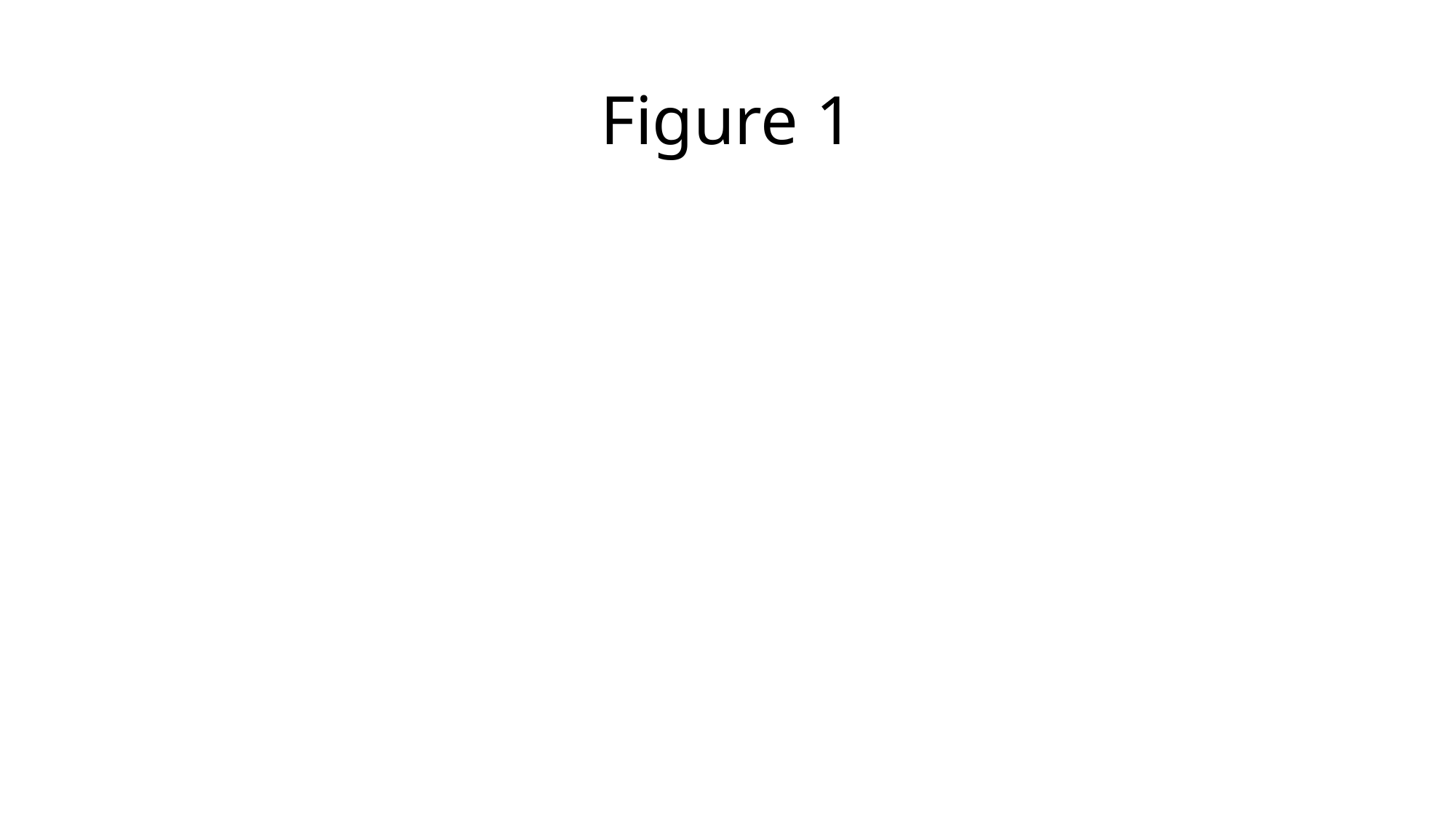

# Figure 1

## Slide 2
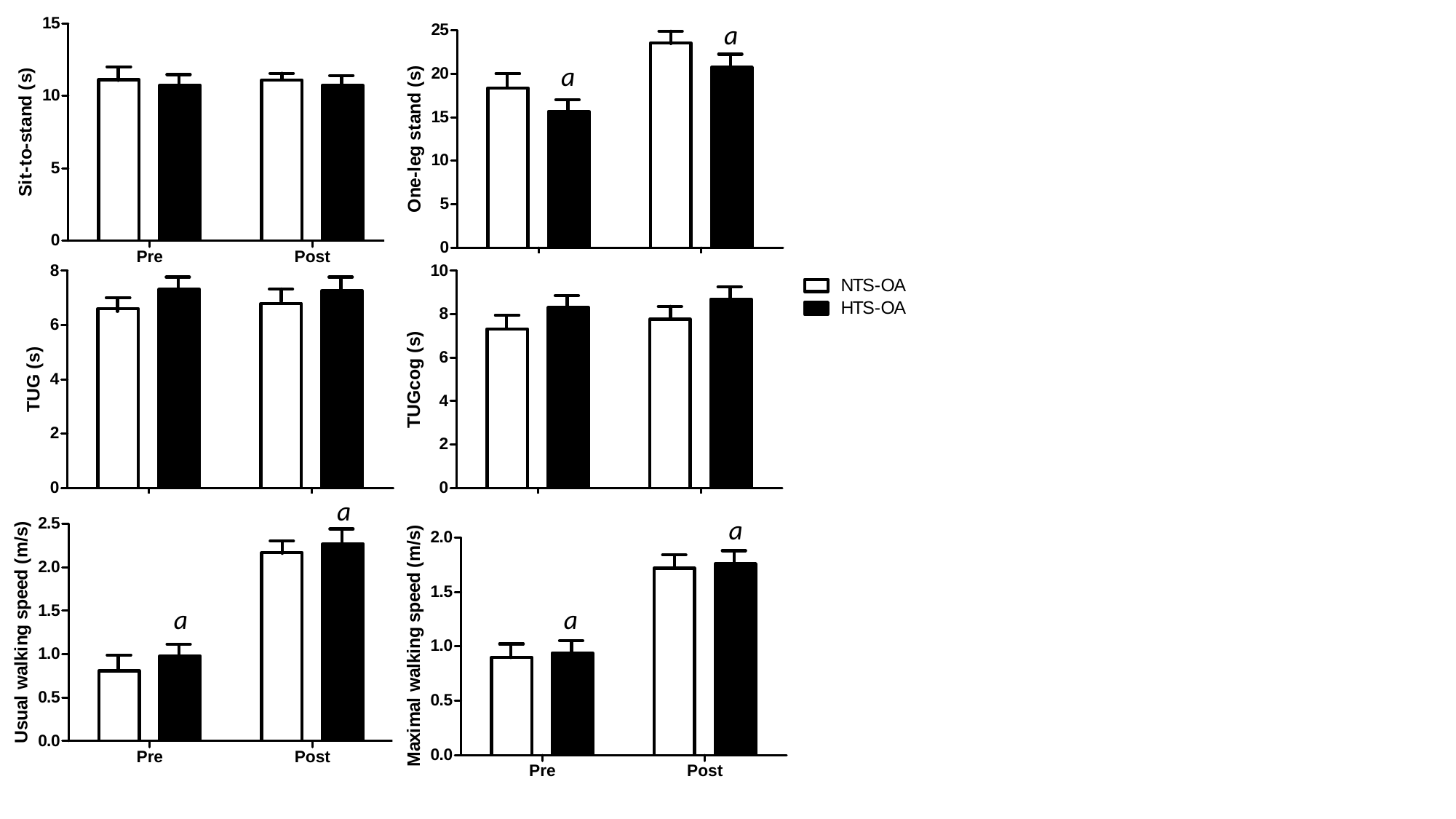

a
a
a
a
a
a

## Slide 3
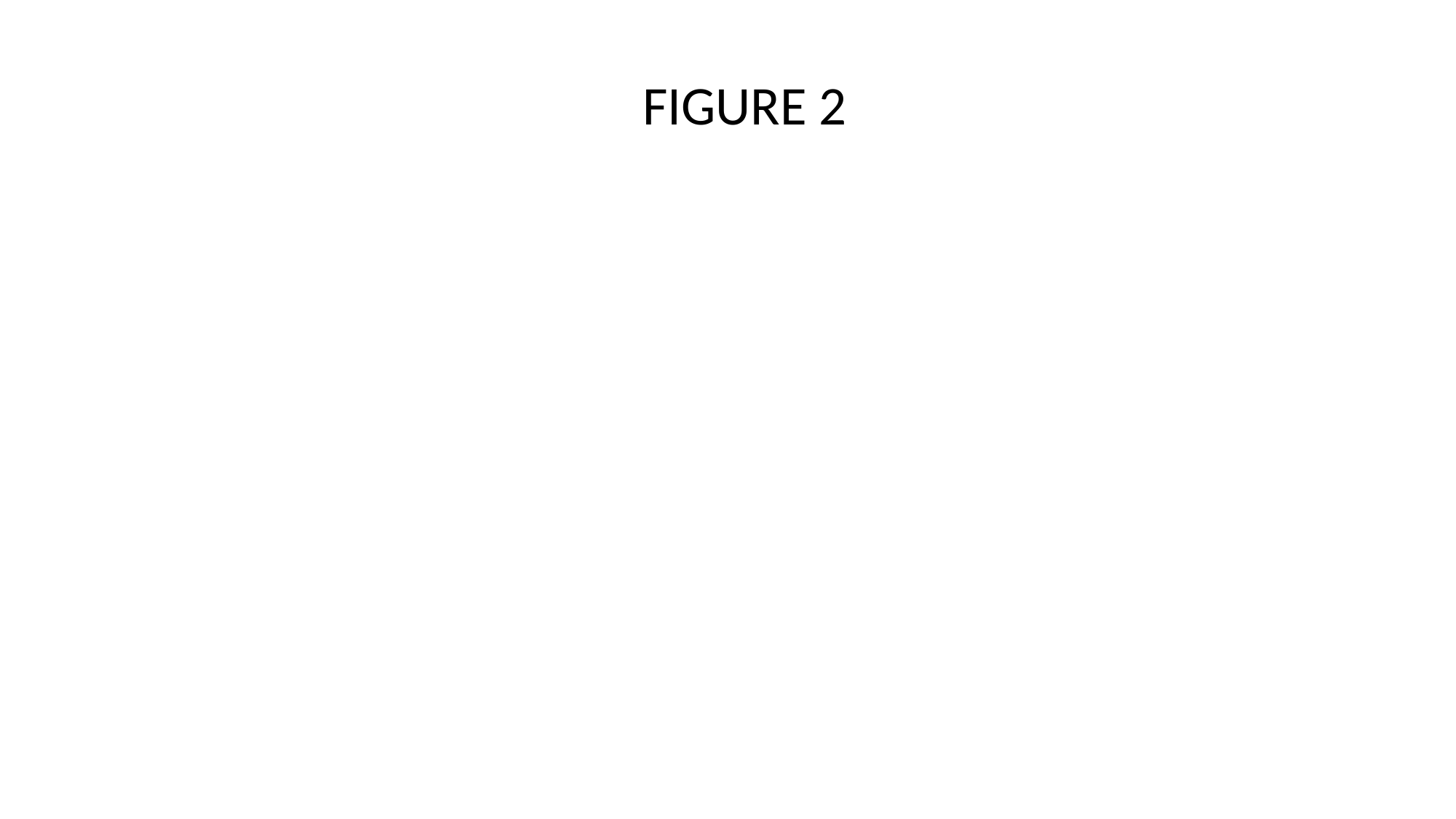

FIGURE 2

## Slide 4
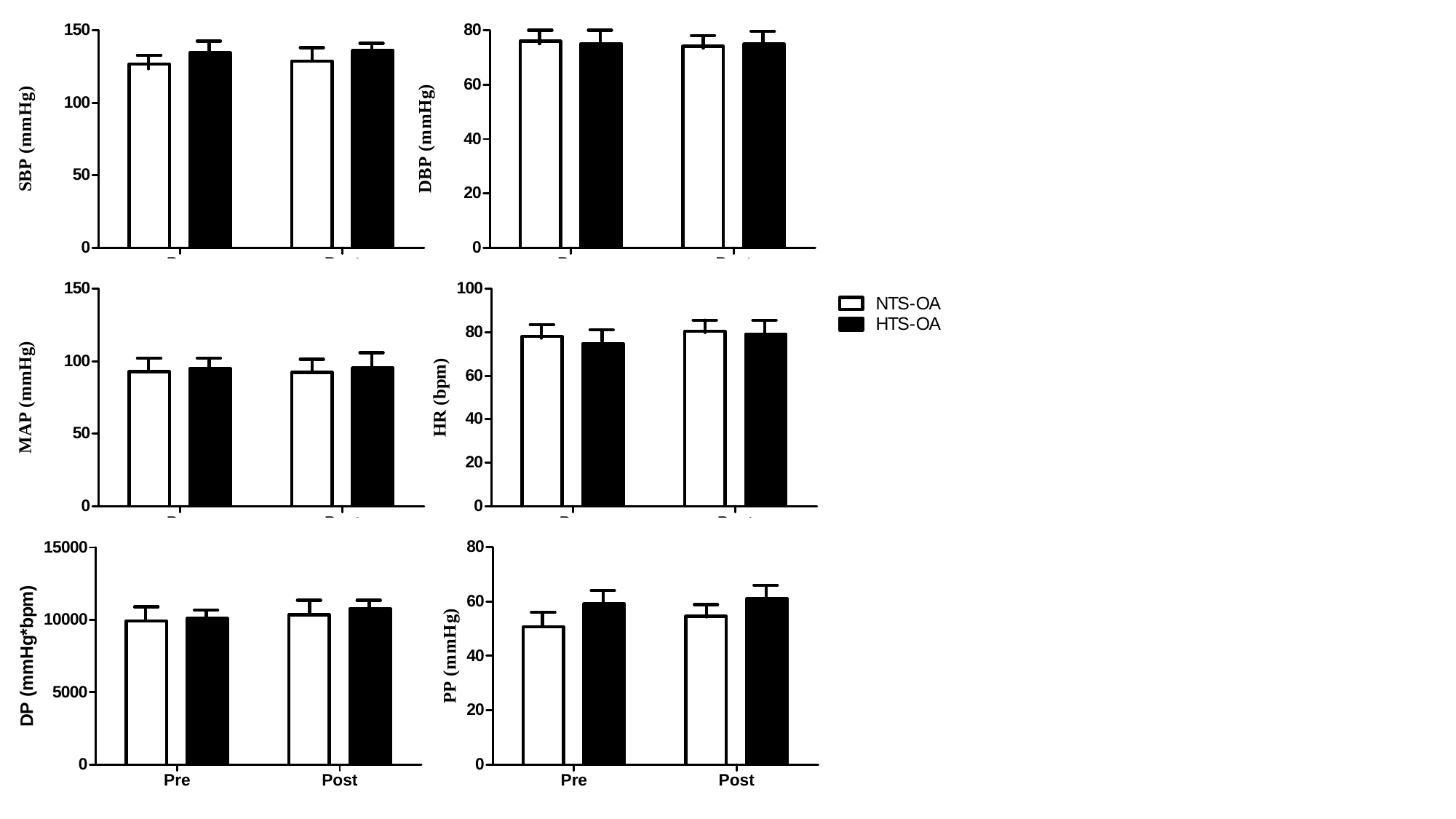

Supplement: Supplementary Materials — may be observed in Figures SM1 and SM2. Figure SM1: functional and cognitive parameters. Data are presented as mean ± SD; NTS: normotensive; HTS: hypertensive; TUG: timed up and go; αP < 0.05 versus pre. Figure SM2: hemodynamic parameters. Data are presented as mean ± SD; NTS: normotensive; HTS: hypertensive; SBP: systolic blood pressure; DBP: diastolic blood pressure; MAP: mean arterial pressure; HR: heart rate; DP: double product; PP: pulse pressure; αP < 0.05 versus pre. [file 3714739.f1.pptx]
